# What Fosters Leaders’ Health Role Modeling? Communication and Remote Work as Boundary Conditions

**DOI:** 10.3390/bs16050827

**Published:** 2026-05-20

**Authors:** Lene S. Fröhlich, Annika Krick, Jörg Felfe, Sarah Kirschnereit, Anna Ernsting

**Affiliations:** 1 Department of Work, Organizational and Economic Psychology, Helmut Schmidt University Hamburg/University of the Federal Armed Forces, 22043 Hamburg, Germany; krick@hsu-hh.de (A.K.); felfe@hsu-hh.de (J.F.); s.kirschnereit@hsu-hh.de (S.K.); 2 Boehringer Ingelheim Pharma GmbH & Co. KG, 55216 Ingelheim am Rhein, Germany; anna.ernsting@boehringer-ingelheim.com

**Keywords:** health-oriented leadership, role model, SelfCare, disclosure, working from home, informal communication

## Abstract

Based on Social Learning Theory, the Health-oriented Leadership Model posits leaders as SelfCare role models. While this modeling influence is established for general SelfCare, its applicability to sensitive behaviors—such as disclosing mental health problems—remains unclear. Additionally, the role of interactional and contextual factors is insufficiently understood. The present paper investigates whether leaders’ role modeling extends to disclosure and whether communication aspects and Working from Home (WfH) intensity moderate these effects. Two cross-sectional studies were conducted among employees working partly from home. Employees rated their own and leaders’ SelfCare; Study 2 (pharmaceutical company; *N* = 198) additionally assessed disclosure. Both studies included communication frequency and WfH intensity; Study 1 (public service; *N* = 227) measured informal communication, and Study 2 assessed communication barriers. Results confirmed that leaders’ SelfCare and disclosure were related to employees’ corresponding behaviors. Communication frequency and WfH intensity showed no moderating effects. Informal communication was associated with a stronger leader SelfCare role model effect, whereas communication barriers were associated with weaker role model effects. Findings suggest an association between leaders’ and employees’ health behavior, consistent with role modeling processes. Based on these preliminary findings, organizations may raise leaders’ awareness of their impact, while leaders should hold informal check-ins and promote barrier-free communication. Future longitudinal and experimental research should validate these findings.

## 1. Introduction

Workplace health promotion is becoming increasingly relevant in public health and organizational contexts (World Health Organization, 2022). Organizations and leaders must adopt targeted strategies to protect employee health and maintain performance, ensuring long-term success in terms of productivity, reduced sickness absence, and employee retention (e.g., De Oliveira et al., 2023; Grinza & Rycx, 2020). Leaders play a pivotal role in fostering and supporting employee health (e.g., Montano et al., 2023). In this regard, research on the concepts of Health-oriented Leadership (HoL; Franke et al., 2014) and health-promoting leadership (e.g., Jiménez et al., 2017) has demonstrated that leaders can positively influence employees across various work-related domains, including health and engagement (Liu et al., 2022), exhaustion (Grimm et al., 2021), as well as task performance and innovative work behavior (Zhang & Liu, 2022). According to the HoL Model (Franke et al., 2014), leaders may not only create health-promoting working conditions and actively support employees’ health, but also act as role models for health-oriented behavior (i.e., SelfCare; Franke et al., 2014). By taking care of their own health and thus modeling SelfCare (e.g., getting enough relaxation and recovery, taking part in occupational health promotion activities, reducing demands by optimizing the personal work routine, working conditions, and work–life balance), leaders can set normative examples within the organizational culture (Wegge et al., 2014), thereby encouraging and supporting their followers to do the same. Based on Social Learning Theory (Bandura, 1969; Bandura, 1971) and Social Cognitive Theory (Bandura, 1986) such role modeling can lead employees to adopt similar SelfCare behaviors through observational learning.

While leaders’ role model effects have been demonstrated for SelfCare (Klug et al., 2019) and various specific health-related behaviors such as presenteeism (Dietz et al., 2020) and the maintenance of work–life boundaries (A. R. Koch & Binnewies, 2015), it is unclear whether this dynamic also extends to a particularly critical form of SelfCare: the disclosure of mental health problems or psychological strain. Unlike more commonly discussed forms of SelfCare (e.g., nutrition, exercise), disclosure involves openly communicating vulnerability (Ragins, 2008), making it an important gateway to organizational intervention and leader support (Pischel et al., 2022). On the one hand, it is conceivable that leaders openly discussing their psychological strain may help normalize mental health conversations and reduce fear of negative consequences, encouraging employees to disclose their psychological strain as well. On the other hand, leader disclosure may prove insufficient as employees may perceive a lack of similarity to their higher-status leaders, while deeply rooted negative biases may also leave stigma perceptions and consequent non-disclosure largely unchanged. Investigating whether employees emulate leaders’ disclosure behavior through role modeling is therefore essential to determine whether such openness serves not only as an effective and credible way for leaders to actively promote employee health (Greenberg & Stone, 1992) but also as a means of fostering a team climate that prioritizes mental health.

However, contextual and interpersonal boundary conditions may be associated with the emergence of the role-model effect for SelfCare and the disclosure of mental health problems. We argue that informal communication between leader and follower may be particularly crucial for the role model effect. While communication has gained increasing recognition as a general component of effective leadership (e.g., Omilion-Hodges & Baker, 2017; De Vries et al., 2010; Yue et al., 2021), it is yet unknown whether communication aspects are associated with the extent to which leaders function as health-related role models. Communication aspects can be differentiated in terms of their quantity, quality, and content (Marlow et al., 2017), referring respectively to how often communication occurs, how communication is experienced (e.g., misunderstandings, complicated communication), and what is being communicated (e.g., task-related or informal communication). Informal, non-work-related communication involves spontaneous, personal exchanges in the workplace outside structured processes (e.g., T. Koch & Denner, 2022; Kraut et al., 1990). Unlike the relevance of communication quantity and quality (e.g., Marlow et al., 2018; Shockley et al., 2021), informal communication as a content aspect was long considered irrelevant or even detrimental to organizational effectiveness (e.g., Tautz et al., 2023), and has only recently been highlighted through research (e.g., Fay & Kline, 2011; T. Koch & Denner, 2022).

Drawing from Social Learning Theory (Bandura, 1969), we argue that communication quantity (i.e., communication frequency), quality (i.e., perceived communication barriers), and content (i.e., informal communication) are all associated with leaders’ health-related role-modeling. Frequent, high-quality, as well as informal communication may enhance the visibility and salience of leaders’ health behaviors, thereby increasing the likelihood that employees will emulate them (Bandura, 1969, 1971, 1986). More precisely, regarding informal communication, it may help leaders to better understand employees’ needs, competencies, and personal backgrounds, which is crucial for employee-centered leadership styles (Tautz et al., 2022). It conveys social cues, fosters shared norms (Fay & Kline, 2011), and supports interpersonal relationships at work (Holmes & Marra, 2004), which may promote trust in and identification with the leader—key conditions for role model perception (Bandura, 1969, 1971, 1986; Bass & Steidlmeier, 1999). Conversely, poor communication between leaders and employees—characterized by communication barriers such as misunderstandings and lack of clarity—may hinder mutual understanding, impede relationship building, and weaken the role model effect.

Related to communication aspects, a contextual factor may also play a crucial role in the emergence of leaders’ health-related role model effect. Although remote and hybrid work is continuously growing (Eurostat, 2022), their impact on the role model effect remains understudied (Krick & Felfe, 2022). Remote and hybrid work reduces social cues and face-to-face interactions, potentially limiting opportunities to observe leaders’ SelfCare behavior or reducing opportunities for disclosure. Social learning mechanisms such as attention and identification (Bandura, 1969, 1971, 1986) may be impaired when the Working from Home (WfH) intensity is high, diminishing the perception of leaders as authentic role models (Eisenberg et al., 2019). Understanding if WfH shapes the dynamic of the role model effect is important for leaders and organizations to proactively shape workplace conditions to enhance health-related role modeling, rather than leaving this potential untapped.

We conducted two studies to address these research gaps by strengthening the empirical basis for the SelfCare role model effect of leaders and extending it to the domain of disclosure. Specifically, we examined if leaders’ disclosure of mental health problems encourages employees to disclose their strain in return. In our first study, we examined how interactional factors (communication frequency and informal communication) and the contextual factor of WfH intensity are associated with leaders’ SelfCare role model effect. Our second study replicated and extended these findings in the context of disclosure, additionally considering communication barriers as a potential limiting factor in the transmission of leaders’ health-related role model effects.

With these studies, we aim to contribute to several areas of research: First, our findings add to the still limited but growing body of evidence supporting the role model pathway in the HoL Model (Franke et al., 2014). We not only replicate the role model effect for general SelfCare but also provide first cross-sectional indications that the effect may extend to the disclosure of psychological strain or mental health problems, representing a more sensitive form of SelfCare. Second, we advance existing disclosure models by suggesting role modeling as an antecedent (e.g., Ragins, 2008), based on initial cross-sectional evidence. Third, we expand to both general and leadership-specific frameworks, such as the Social Learning Theory (Bandura, 1969), the Social Cognitive Theory (Bandura, 1986), and the HoL Model (Franke et al., 2014), by identifying contextual and interactional factors that may be associated with the role model effect. In doing so, we respond to calls for more contextual leadership research (Johns, 2024) and for exploring the link between leadership and communication (De Vries et al., 2010). Taking a more differentiated perspective on communication, we address the question of whether informal communication is merely a distraction or whether it may generate beneficial effects in organizational contexts (T. Koch & Denner, 2022). Fourth, we add to the debate on how remote and hybrid work settings may affect employee health (e.g., Beckel & Fisher, 2022; Lunde et al., 2022), specifically by addressing whether remote settings may be associated with weaker leaders’ health-related role model effects (Krick & Felfe, 2022). Finally, these studies may provide initial cross-sectional indications of why previous research (e.g., Klug et al., 2019) found only a moderate link between leaders’ and employees’ health behavior by pointing to contextual and interactional factors that may be associated with the strength of the role model effect. Practically, our findings may help generate hypotheses for future longitudinal and experimental research and provide organizations with preliminary indications for supporting leaders in using communication strategically to model health-promoting behaviors more effectively. Furthermore, by providing initial cross-sectional evidence on whether and how remote work contexts are associated with leaders’ health-related role model effect, our studies offer a starting point for future research to follow up on, with potential implications for how organizations design and implement their remote work policies.

## 2. Leaders as Behavioral Models

The Social Learning Theory (Bandura, 1969) suggests that individuals can acquire new behaviors by observing others. Bandura (1986) later expanded this concept into the Social Cognitive Theory, emphasizing that cognitive factors such as attention, retention, and motivation within the observer determine whether a behavior is emulated. This perspective also explains which attributes of both the observer and the model influence whether someone is perceived and adopted as a role model. In their Motivational Theory of Role Modeling, Morgenroth et al. (2015, p. 468) define role models as “individuals who influence role aspirants’ achievements, motivation, and goals by acting as behavioral models, representations of the possible, and/or inspirations”. In organizational settings, leaders are particularly likely to be perceived as role models, since individuals tend to imitate the behavior of those with admired status (Bandura, 1969). Within the workplace, leaders shape both explicit and implicit norms and standards for acceptable and valued behavior (Wegge et al., 2014). Within the concept of Transformational Leadership, serving as a role model is conceptualized as a central explanatory mechanism of leaders’ influence on followers’ motivation and behavior (Bass & Avolio, 1994a, 1994b).

## 3. Leaders’ Role Modeling of Health-Oriented Behavior

The role-modeling function of leaders extends beyond task-related or performance-oriented domains. As Transformational Leadership Theory emphasizes, leaders influence followers not only through directives but also by exemplifying values and behaviors that are admired and emulated (Bass & Avolio, 1994a, 1994b). Building on this broader understanding of role modeling, the HoL Model (Franke et al., 2014) suggests that leaders can also shape their employees’ health-related behaviors by acting as role models in this domain. The HoL Model comprises three main components in the workplace: leaders’ and employees’ *SelfCare* (i.e., self-directed health-oriented behavior) and leaders’ *StaffCare* (i.e., employee-directed health-oriented leadership). Leaders’ and employees’ SelfCare and leaders’ StaffCare each comprise three facets: value, awareness, and behavior. When leaders and employees practice SelfCare, they prioritize their own health (value), pay attention to health warning signals in themselves (awareness), and take actions to maintain and promote their health (behavior). Similarly, leaders who demonstrate StaffCare assign high importance to their employees’ health (value), recognize health warning signals and stressors in their employees (awareness), and actively encourage and support them in engaging in health-promoting behaviors (behavior). According to the model, leaders’ SelfCare fosters employees’ SelfCare through the role model effect (Franke et al., 2014).

### 3.1. SelfCare Role Modeling

By demonstrating health-neglecting or health-promoting SelfCare behavior, a leader can set a benchmark for the value placed on health in general within the team and signal which behavior is accepted or at least not punished within the organization (Klug et al., 2019). For example, Hammer et al. (2007) explain that some leaders deliberately avoid sending e-mails on the weekend to prevent employees from thinking that this is an expected behavior. Similarly, leaders could consciously try to be role models in terms of work–life balance, for example, by prioritizing family matters when necessary (Hammer et al., 2007) to encourage employees to also balance work with leisure and rest. Since SelfCare has numerous positive effects on employees, such as improvements in general health (e.g., Klug et al., 2019), well-being (Santa Maria et al., 2019), and work engagement (e.g., Grimm et al., 2021), the promotion of employee SelfCare through leaders’ role modeling appears to be a meaningful objective. Despite its potential relevance, the role model effect of leaders’ SelfCare has received limited empirical attention to date. To our knowledge, only two studies have investigated this effect from the employee’s perspective, identifying a positive relationship between leaders’ and employees’ SelfCare and thus offering support for the existence of a leader-driven SelfCare role model mechanism (Fröhlich et al., 2025; Klug et al., 2019). Drawing on Bandura’s (1969) Social Learning Theory, we refer to the behavioral dimension of SelfCare, which is the easiest to observe by followers. Consistent with the findings of Klug et al. (2019) and Fröhlich et al. (2025), as well as the assumptions of the HoL Model (Franke et al., 2014), we propose the following hypothesis:

**Hypothesis** **1 (H1):** *Leaders’ SelfCare behavior is positively related to employees’ SelfCare behavior.*

### 3.2. Modeling Vulnerability: Disclosure Role Modeling

In addition to general SelfCare behaviors, prior research has provided empirical evidence that leaders serve as health-related role models for presenteeism (Dietz et al., 2020) and work–life balance (A. R. Koch & Binnewies, 2015). Notably, existing studies appear to predominantly focus on socially accepted behaviors. However, it is unclear whether leaders also serve as role models for more sensitive SelfCare behaviors that are important for receiving support but may simultaneously be perceived as involving potential risks or negative consequences. One such behavior is the disclosure of mental health problems or psychological strain. Disclosure describes the act of an employee sharing their mental health problems or psychological strain with their supervisor (Ellison et al., 2003). Individuals experiencing psychological strain or mental health difficulties often face a challenging decision regarding whether to disclose their condition to others (Brohan et al., 2012), as doing so may expose them to potential stigma (Ragins, 2008). Mental health stigma is frequently associated with negative stereotypes, including perceptions of individuals as being incompetent, weak, or potentially dangerous (e.g., Follmer et al., 2020; Krupa et al., 2009; Toth & Dewa, 2014). Drawing on Social Identity Theory (SIT; Tajfel & Turner, 2004), Toth and Dewa (2014) emphasize that stigma can contribute to a discredited personal social identity for those affected in collective contexts such as the workplace. To avoid negative consequences, individuals often base their decision to disclose on a cost–benefit analysis of anticipated outcomes (Ragins, 2008). Existing disclosure models identify a range of antecedents, such as supportive relationships, perceived organizational and leader support, and perceived job security (Jones & King, 2014; Ragins, 2008; Toth & Dewa, 2014). Recent research also found that HoL is positively associated with employee disclosure (Pischel & Felfe, 2023). StaffCare not only functions as an antecedent to employee disclosure, but can also be a positive outcome arising from employees’ openness about their mental health problems, underscoring a bidirectional relationship between leaders’ StaffCare and disclosure (Pischel et al., 2022). Additional positive consequences of disclosure include increased well-being and authenticity (Corrigan et al., 2013). Conversely, nondisclosure may have detrimental effects on employees, such as exacerbated stress and insufficient support, as supervisors might remain unaware of the underlying issues and thus be unable to provide adequate assistance (Jones & King, 2014; Ragins, 2008). In some instances, leaders may even unintentionally increase pressure by misattributing reduced performance to other causes (Martinko et al., 2007). Therefore, disclosing mental health problems presents a significant dilemma for employees: While it involves personal risk for already psychologically strained individuals, it also holds the potential for beneficial outcomes such as support and relief. This duality renders disclosure a particularly critical and complex SelfCare behavior. The findings by Pischel and Felfe (2023) underline the role of leaders as key drivers of employee disclosure behavior, demonstrating that StaffCare is positively related to employee disclosure. However, it is unclear whether leaders can also foster employees’ disclosure by disclosing their own mental health problems or psychological strain through the role model effect.

On the one hand, employees might not identify sufficiently with their leaders due to status differences, which could limit perceived similarity and thus hinder the emergence of a meaningful role model effect (Bandura, 1969), ultimately resulting in continued reluctance to disclose. On the other hand, SIT (Tajfel & Turner, 2004) provides a theoretical framework for this potential role-modeling process: Individuals derive part of their self-concept from their group memberships and are motivated to maintain a positive social identity in the workplace. When leaders openly acknowledge and manage mental health problems, they may signal that disclosure is socially accepted and not detrimental to one’s identity within the group. This may be associated with reduced perceived stigma and fear of negative consequences, potentially contributing to a climate of trust and psychological safety, which may in turn encourage employees to disclose as well. This assumption is also supported by empirical evidence that shows that disclosure by one individual in a dyadic social relationship tends to elicit reciprocal disclosure from the other party (Cozby, 1973). More specifically, regarding the supervisor-student relationship, Martinez et al. (2014) showed in a medical healthcare setting that supervisors failing to disclose medical errors or reacting punitively to colleagues’ disclosures inhibited such disclosure behavior in students. In addition, it can be assumed that leaders who share vulnerability foster stronger emotional connections with employees (Ito & Bligh, 2016), which may encourage employees to share similar vulnerabilities in turn. In light of these reflections and findings, we propose that the leaders’ health-related role model effect of the HoL Model (Franke et al., 2014) can be extended to the critical aspect of SelfCare in the form of disclosing mental health problems. We propose the following hypothesis:

**Hypothesis** **2 (H2):** *Leaders’ disclosure of mental health problems or psychological strain to their employees is positively related to employees’ disclosure of mental health problems or psychological strain to their leaders.*

### 3.3. Boundary Conditions of Leaders’ Health-Related Role Model Effect

The Social Learning Theory (Bandura, 1969) explains how individuals adopt role models by observing and imitating their behavior through processes of *attention*, *retention*, *reproduction*, and *motivation*. Additionally, factors related to the observer (e.g., self-efficacy), the model (e.g., status), and their relationship (e.g., identification and perceived similarity) influence the likelihood of imitation (Bandura, 1969, 1971, 1986). Thus, imitation is not automatic or universal, as it depends on a set of individual, interactional, and contextual preconditions. While these general mechanisms are well established, it remains unclear which specific factors facilitate or hinder leaders’ role modeling of health-related behaviors, such as SelfCare and disclosure of mental health problems. To our knowledge, only Fröhlich et al. (2025) have examined factors that influence whether individuals can benefit from a positive SelfCare role model and resist a negative SelfCare example. By using a person-centered approach, they found, for instance, that leaders’ StaffCare positively supports both mechanisms—enhancing the likelihood of benefiting from a positive role model and resisting a negative one—whereas employees’ stress vulnerability negatively affects both, making individuals less likely to benefit from positive examples and more susceptible to negative ones (Fröhlich et al., 2025). Drawing on Social Learning Theory (Bandura, 1969, 1971), Social Cognitive Theory (Bandura, 1986), and Social Information Processing Theory (SIP; Salancik & Pfeffer, 1978), we argue that both direct interpersonal communication and the broader communication context—specifically in terms of WfH—may be associated with leaders’ health-related role model effect.

Leadership and communication are inherently intertwined, as leadership is a communication-based process (Johnson & Hackman, 2018) and leaders exert influence on their employees through communication (Eisenberg et al., 2019). The SIP Theory (Salancik & Pfeffer, 1978) posits that employees’ perceptions and behaviors in the workplace are shaped by social cues from the environment, signaling which behaviors are valued. These cues can be transmitted through leaders’ communication, making certain SelfCare behaviors or their positive consequences more visible and salient and increasing opportunities for their disclosure. Thus, referring back to Social Cognitive Theory (Bandura, 1969, 1971, 1986), the way leaders and employees communicate may increase the likelihood of imitation.

Based on Marlow et al.’s (2017) framework of communication processes in virtual teams, communication includes quantity (i.e., communication frequency), quality (e.g., communication barriers), and content (e.g., formal vs. informal communication). More frequent, high-quality, and informal communication may enhance the salience of leaders’ role model behaviors. Communication also fosters trust and relationship quality (Omilion-Hodges & Baker, 2017; Schoorman et al., 2007), which may enhance perceptions of authentic role models and increase the likelihood of imitation.

While interpersonal communication may be a key mechanism of leaders’ health-related role modeling, its effectiveness may vary across broader work contexts which should therefore be considered as an additional boundary condition. One significant contemporary contextual factor is the intensity of WfH. Although WfH strongly influences communication by reducing face-to-face (F2F) exchanges (e.g., Gajendran & Harrison, 2007; Lal et al., 2021), its impact extends beyond communication alone. Increased physical distance can alter how leaders’ behaviors are perceived in virtual versus in-person settings. These changes may affect the extent to which leaders are viewed as authentic, inspiring, and credible role models (e.g., Eisenberg et al., 2019; Shamir & Ben-Ari, 1999). Thus, WfH intensity should be considered as a broader contextual condition that may constrain the role model effect.

From a resource-oriented perspective, communication and the context correspond with the Job Demands–Resources (JD-R) Model (Demerouti et al., 2001; Bakker & Demerouti, 2007, 2017). The JD-R Model (Demerouti et al., 2001) conceptualizes job characteristics either as resources or demands. Job demands are those physical, psychological, social, or organizational aspects of the job that require sustained effort and are therefore associated with certain costs (e.g., stress, reduced well-being; Bakker & Demerouti, 2007, 2017). In contrast, job resources refer to those aspects of the job that help achieve work goals, reduce job demands and their associated costs, and foster work engagement, motivation, and well-being (Bakker & Demerouti, 2007, 2017). Thus, applied to the present context, frequent and informal communication may be conceptualized as a job resource: It may increase behavioral visibility, strengthen trust and relationship quality, and support the leader’s role model function by facilitating employees’ social learning. Conversely, high WfH intensity and communication barriers may represent job demands, as they may reduce opportunities for spontaneous interaction, constrain the observation of leaders’ behaviors, and impede imitation processes. In the following sections, we conceptualize communication aspects and WfH intensity as moderators affecting leaders’ role model effects regarding SelfCare and the disclosure of mental health problems.

#### 3.3.1. Communication Frequency: A Driver of Role Model Visibility?

*Communication quantity* or *frequency* is defined as the overall volume of exchanges through various communication channels (Marks et al., 2000; Marlow et al., 2017). Much of prior research has focused on the direct effects of communication frequency on performance (e.g., Marlow et al., 2018; Shockley et al., 2021). This raises the question of whether communication frequency may also be associated with the relationship and dynamic between leaders and followers.

On the one hand, overly frequent communication could overwhelm employees, reducing message salience and diluting the visibility of leaders’ SelfCare behaviors or disclosures. This argument aligns with Shockley et al.’s (2021) assumption that too much communication may even contribute to burnout symptoms. Although this was not empirically supported (Shockley et al., 2021), the broader notion that too much communication frequency may be counterproductive remains plausible. On the other hand, from a JD-R perspective (Bakker & Demerouti, 2007, 2017), frequent communication may be considered a job resource that enables other resources, such as leaders’ health-related role model effects. Several arguments can be made in favor of this: First, as a mechanism of the role model effect, the emulation of a behavior requires repeated and visible exposure (Bandura, 1969). Frequent communication increases the opportunities for leaders to make their SelfCare behaviors salient, even when employees do not directly observe them. For example, leaders might mention having sought support or taken a break—behaviors that, through communication, become visible and available for imitation. The same may hold for the disclosure of mental health problems or psychological strain. Frequent communication and interactions may create opportunities for leaders and employees to open up. Since disclosing sensitive information already demands considerable courage and involves weighing emotional and social risks (e.g., Brohan et al., 2012; Ragins, 2008), frequent communication may facilitate such openness in the first place. Bruhn et al. (2025) additionally showed that communication frequency positively predicted perceptions of StaffCare, suggesting that more frequent exchanges may also enhance leaders’ visibility and, consequently, also their health-related role model function.

Second, the role model effect depends on the relationship between the model and the observer, particularly the identification with the model (Bandura, 1969). The perceived relationship is also crucial for disclosing psychological strain or mental health problems (Greene, 2009). Frequent communication fosters trust (Jarvenpaa & Leidner, 1999), which in turn can enhance the quality of the leader-employee relationship. This strengthened relationship quality may further reinforce the leader’s role model function concerning SelfCare and disclosure. Conversely, when contact is infrequent, employees are more likely to perceive a sense of distance from their leaders (Antonakis & Atwater, 2002), potentially reducing the perceived relevance of or similarity with the leader and thus undermining the leader’s role model potential (Bandura, 1969, 1971).

Overall, it is reasonable to postulate that frequent communication may be associated with a stronger leaders’ role model effect by increasing their visibility, relational closeness, and the potential for observational learning. Drawing on the arguments outlined above, we further propose the following hypotheses:

**Hypothesis** **3a (H3a):** *Communication frequency moderates the relationship between leaders’ SelfCare behavior and employees’ SelfCare behavior. The relationship is stronger at higher levels of communication frequency (Study 1 + 2).*

**Hypothesis** **3b (H3b):** *Communication frequency moderates the relationship between leaders’ disclosure and employees’ disclosure. The relationship is stronger at higher levels of communication frequency (Study 2).*

#### 3.3.2. Communication Barriers: Obstacles for Role Modeling?

*Communication barriers* refer to obstacles or conditions that hinder the accurate transmission and interpretation of information by distorting the message, causing misunderstandings, or preventing key aspects from being recognized by the receiver (Daud et al., 2017; Kamal Bahrain et al., 2023). In the framework by Marlow et al. (2017), communication barriers can be understood as part of *communication quality*, which is defined as “Clarity, effectiveness, accuracy, and completeness of communication” (Marlow et al., 2017, p. 577; González-Romá & Hernández, 2014). Thus, barriers that hinder these aspects reduce overall communication quality. According to Marlow et al. (2017), the relevance of communication quality even exceeds that of communication frequency, as it contributes more directly to the development of “shared understanding” between the involved parties (Marlow et al., 2017, p. 578). Empirical findings demonstrate positive effects of high-quality communication on job performance and burnout reduction (Shockley et al., 2021). Bruhn et al. (2025) also reported that perceived communication barriers were negatively associated with StaffCare, suggesting that obstacles in communication can weaken leaders’ health-related influence on their employees.

Given the inherent dependence of leadership on communication (Johnson & Hackman, 2018) and building on the JD-R model (Demerouti et al., 2001), high-quality communication may function as a job resource that enhances other resources, such as leadership effectiveness, whereas communication barriers, conceptualized as job demands, may constrain them. Indeed, Tautz et al. (2024) demonstrated that poor communication, conceptualized as a job demand (Day et al., 2012; Stich et al., 2015), attenuates the positive effect of Transformational Leadership on employee well-being. Therefore, leaders’ role model function—a key component of Transformational Leadership (Bass & Avolio, 1994a)—may likewise be impaired by communication barriers. Accordingly, such barriers may limit the extent to which leaders can effectively serve as role models for SelfCare and disclosure. This pattern may be understood through three explanatory pathways.

First, when employees experience communication with their leader as complicated, ambiguous, or prone to misunderstandings, this may impair their perception of the leader’s trustworthiness (Tautz et al., 2024), as mutual understanding is lacking. Thus, communication barriers may weaken the quality of the leader–employee relationship or create a sense of disconnection (Kamal Bahrain et al., 2023), potentially diminishing the perception of the leader as a credible and influential role model. Therefore, even if a leader models SelfCare behaviors or discloses mental health problems, communication barriers may reduce employees’ willingness to imitate such behaviors or to respond with reciprocal openness.

Second, Kamal Bahrain et al. (2023, p. 1495) highlight that the effort required to overcome communication barriers is perceived as “stressful and unpleasant for employees”. Such barriers can be viewed as job demands that drain mental and emotional resources. Employees may have reduced capacity to engage in SelfCare or disclose mental health problems, further limiting the role model effect.

Third, there is also a cognitive explanation for why communication barriers may hinder behavioral imitation: If leaders’ health-related behaviors are communicated in a vague, ambiguous, or nonspecific manner, employees may struggle to understand or translate them into concrete action. Observational learning is most effective when the modeled behavior is clear, relevant, and actionable (Bandura, 1969, 1971, 1986). When communication lacks clarity, employees may misinterpret the behavior or miss the intended message entirely. Regarding disclosure, this ambiguity may fail to signal that disclosing mental health problems is acceptable or safe, thereby reducing the potential for reciprocal openness or behavioral imitation. Based on the foregoing reasoning, we propose the following hypotheses:

**Hypothesis** **4a (H4a):** *Communication barriers between leaders and employees moderate the relationship between leaders’ SelfCare behavior and employees’ SelfCare behavior. The relationship is weaker at higher levels of communication barriers (Study 2).*

**Hypothesis** **4b (H4b):** *Communication barriers between leaders and employees moderate the relationship between leaders’ disclosure and employees’ disclosure. The relationship is weaker at higher levels of communication barriers (Study 2).*

#### 3.3.3. Informal Communication: A Catalyst for the SelfCare Role Model Effect?

*Informal communication* refers to non-work-related, spontaneous, and personal communication between individuals within the workplace that occurs outside of formal structures and without the intention to address work-related tasks. It typically takes place between individuals who are not interacting in their professional roles, but rather in a social or personal role (e.g., T. Koch & Denner, 2022; Kraut et al., 1990). From a conceptual standpoint, informal communication can be situated within the *content* dimension of communication, reflecting a “relational-oriented” content rather than a “task-oriented” one (Marlow et al., 2017, p. 577). Since informal communication was long considered to be irrelevant or even detrimental to an organization’s effectiveness (Tautz et al., 2023), previous research on communication in organizations has instead focused on formal communication (Viererbl et al., 2022). However, empirical findings emphasize that informal communication yields various organizational benefits (Enyia & Orokor, 2016). Through promoting affective commitment and job satisfaction, it can enhance employee productivity (Methot et al., 2021). T. Koch and Denner (2022) further highlight that informal communication provides an outlet for frustration and anger, builds trust, develops relationships (Conrad & Poole, 2012; Holmes & Marra, 2004; T. Koch & Denner, 2022), fosters connection and mutual understanding (Fay, 2011), creates person-centered knowledge, and enhances coworker liking (Fay & Kline, 2011). In contrast, formal communication is generally considered unsuitable for sharing private or sensitive information (Conrad & Poole, 2012). Given these positive interpersonal and relational effects, Tautz et al. (2023) argue that informal communication may also enhance leadership effectiveness. They conclude that informal exchanges can be a valuable leadership tool and should be actively encouraged by leaders to strengthen relationships and foster a supportive team environment. To date, it remains unclear whether informal communication can also enhance leaders’ health-related role model effect. However, given its key role in transmitting organizational culture by conveying implicit social norms, shared values, and behavioral expectations (Fay & Kline, 2011), informal communication may likewise reinforce leaders’ SelfCare role model effect. Two main arguments support this assumption.

First, as previously noted, informal interactions between leaders and employees foster getting to know each other and the development of interpersonal relationships (e.g., Fay & Kline, 2011; Holmes & Marra, 2004; T. Koch & Denner, 2022). Such relational closeness is a fundamental prerequisite for identification with the leader (Kelman, 1958), which increases the likelihood that employees view the leader as a trustworthy role model (Bandura, 1969). Lütjens and Felfe (2025) argue that informal communication also reduces hierarchical distance and highlights shared values. They found empirical support for the positive impact of informal communication on employees’ perceptions of Transformational Leadership (Lütjens & Felfe, 2025). Since Transformational Leadership also relies on visibility and authenticity to influence followers, these same mechanisms may similarly support leaders’ health-related role model effect. Together, these relational and perceptual mechanisms may make employees more receptive to adopting their leaders’ health-related behaviors, including SelfCare.

Second, informal communication fosters the sharing of person-centered knowledge (Fay & Kline, 2011) and provides a natural context for exchanging personal information (Conrad & Poole, 2012), such as health-related routines. Through these informal interactions, leaders can make their SelfCare behaviors more salient to employees or, when such behaviors are not directly observable, convey this information indirectly. For example, during casual conversations with an employee, a leader may mention prioritizing time with family over work or participating in a workplace health course, thereby enhancing the salience of their SelfCare behaviors. This increased behavioral visibility—whether through direct observation or informal communication—may raise the likelihood of imitation (Bandura, 1969, 1971). Consistent with this, Bruhn et al. (2025) found that informal communication increased employees’ perception of StaffCare, underlining its importance for leaders’ influence on employees’ health-related behaviors. Based on the JD-R perspective (Bakker & Demerouti, 2007, 2017), we propose that informal communication between leaders and employees acts as a job resource in the context of health-related role modeling, enhancing the likelihood of benefiting from leaders’ SelfCare role model effect. We propose the following hypothesis:

**Hypothesis** **5 (H5):** *Informal communication between leaders and employees moderates the relationship between leaders’ SelfCare behavior and employees’ SelfCare behavior. The relationship is stronger at higher levels of informal communication (Study 1).*

#### 3.3.4. Working from Home Intensity: A Challenge for Leaders’ Role Model Effect?

With regard to the communication context, we argue that the *WfH intensity* may also play a role in leaders’ health-related role model effect. The prevalence of individuals working from home has increased in recent years (Eurostat, 2022) especially after the COVID-19 pandemic forced organizations to adapt work arrangements to reduce employees’ F2F interactions. Until now, many different terms and concepts such as *remote work*, *telework*, *telecommuting*, and *WfH* have been used to describe work arrangements where individuals work from locations other than traditional offices (e.g., Nakrošienė et al., 2019). For clarity, we will refer exclusively to WfH in the following, excluding other locations outside the primary workplace or home (e.g., public transportation, cafés). The intensity of WfH can vary as some employees work full-time from home through continuous use of digital communication tools, while others do so part-time (Gajendran & Harrison, 2007; Nakrošienė et al., 2019). The effects of WfH on employees’ health can also vary: While it may offer certain advantages (e.g., through increased flexibility), it can also pose health risks (e.g., Zoom fatigue; Felfe et al., 2022). However, to date, it remains an open question whether WfH intensity may also strengthen or weaken leaders’ health-related role model effect (Krick & Felfe, 2022).

On the one hand, one may argue that WfH intensity may strengthen leaders’ role model effect in terms of the disclosure of mental health problems. The digital distance associated with remote work may reduce immediate social pressure and the sense of being directly scrutinized by others, potentially making it psychologically easier for some individuals to disclose sensitive topics. In addition, the perceived control over the situation—such as the possibility to communicate asynchronously via email or chat—allows individuals more time to carefully choose their words and manage the interaction, possibly further increasing the sense of safety. Supporting this notion, qualitative research from the medical context has shown that remote consultations can help engage hesitant or vulnerable patients, as some find it easier to address sensitive topics at a distance than in F2F encounters (Norberg et al., 2025). However, this effect is context- and person-dependent and has been observed primarily in the doctor-patient relationship. Another potential mechanism facilitating disclosure in remote work settings is that leaders may appear more authentic and relatable when seen in private video call settings, for example, in their home environment. Observing leaders in such a personal context may reduce perceived hierarchical distance and make them more approachable, which in turn may increase the likelihood of perceiving them as role models (Tautz et al., 2022).

On the other hand, it is conceivable that higher WfH intensity (e.g., employees mostly working from home) may weaken leaders’ effectiveness as health-related role models, as F2F interactions are more limited (Daft & Lengel, 1986; Lal et al., 2021). According to Media Richness Theory, communication media differ in their capacity to convey rich information and immediate feedback, with richer media such as F2F contact being more effective for complex and ambiguous messages (Daft & Lengel, 1986). In remote settings, leaders’ SelfCare behaviors and disclosures of mental health problems—which can involve a degree of ambiguity—may be harder for employees to interpret. As nonroutine messages require rich media for effective communication (Yue et al., 2021), important cues for interpersonal sensemaking can be lost.

A further reason is that leaders often struggle to inspire employees in virtual environments (Antonakis & Atwater, 2002), particularly through role modeling (Shamir & Ben-Ari, 1999). The absence of nonverbal cues and opportunities to observe leaders across different situations can make leadership behaviors appear less authentic (Tautz et al., 2022). Idealized Influence, a core element of Transformational Leadership (Bass & Avolio, 1994b), may be harder to convey remotely, and limited F2F contact may diminish perceived authenticity (Eisenberg et al., 2019). Employees report that leaders’ enthusiasm is more contagious in person than online, making it harder to inspire when working from home (Tautz et al., 2022). For example, virtual encouragement to take breaks or improve nutrition may lack impact if the leader’s own behavior is not directly observable. Consequently, health-related behaviors such as SelfCare or disclosure of mental health problems or psychological strain may be less likely to be imitated.

To sum up, WfH may also hinder relationship development and reduce behavioral visibility—both of which are essential for modeling and identification processes. Opportunities to observe leaders’ SelfCare in everyday contexts (e.g., visibly ending the workday or informally sharing experiences during lunch) are reduced. Relationship building is further challenged by the loss of spontaneous encounters and reduced nonverbal cues (Weisband & Atwater, 1999; Yue et al., 2021). Virtual contexts may feel less human and colder (Shamir, 1999), making it harder to build the trust and identification necessary for perceiving leaders as credible role models (Bandura, 1969).

Drawing on the preceding arguments and the JD-R framework (Bakker & Demerouti, 2007, 2017), we suggest that WfH intensity may be considered a job demand, weakening the motivational and positive effects of leaders’ role model function on employees’ SelfCare. Thus, we postulate the following hypothesis:

**Hypothesis** **6a (H6a):** *Employees’ Working from Home (WfH) intensity moderates the relationship between leaders’ SelfCare behavior and employees’ SelfCare behavior. The relationship is weaker at higher levels of employees’ WFH intensity (Study 1 + 2).*

**Hypothesis** **6b (H6b):** *Employees’ Working from Home (WfH) intensity moderates the relationship between leaders’ disclosure and employees’ disclosure. The relationship is weaker at higher levels of employees’ WFH intensity (Study 2).*

**Figure 1:**
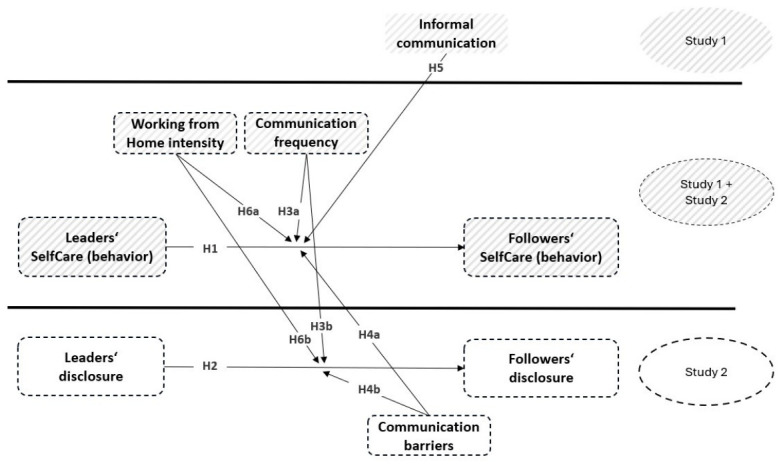
Research model.

Figure 1 illustrates the research model including all hypotheses.

## 4. Study 1

### 4.1. Materials and Methods

#### 4.1.1. Participants and Procedure

To determine the required sample size, an *a priori* power analysis was conducted using G*Power (Version 3.1.9.7; Faul et al., 2009; Heinrich-Heine-Universität Düsseldorf, Düsseldorf, Germany). Based on a medium effect size (f^2^ = 0.15; Cohen, 1988), a significance level of α = 0.05, a power of 1 − β = 0.80, and nine predictors in the regression model, results indicated a minimum sample size of *N* = 114. In early 2025, an online survey was conducted among staff of a public justice administration in Germany, as part of a larger evaluation of telework policies. The survey was administered in German and distributed by a departmental head as well as direct supervisors to all eligible employees, yielding a response rate of 8.72%. As German is the official working language of the institution, all participants can reasonably be assumed to have possessed sufficient German language proficiency to complete the survey. Inclusion criteria were full-time employment and engagement in remote work to some degree, given the telework focus of the study. A total of *N* = 227 employees were included in the final analysis. Before participation, respondents were informed about the voluntary nature of the study and the confidentiality of their data. No missing data occurred for any of the relevant study variables, as all items were set as mandatory in the online survey. The sample consisted of 58.1% males and 41.9% females, with an average age of 44.07 years (*SD* = 10.65). The majority (81.1%) of the participants were employees without leadership responsibility. Educational background varied as follows: 1.8% participants held a doctoral degree, 40.5% a university degree, 26.0% vocational or technical school training, 17.6% a higher secondary school diploma, 11.0% a secondary school diploma, and 3.1% reported a lower secondary school diploma or other educational degree. Participants varied in their WfH intensity: 15.9% worked from home one day per week, 26.9% two days, 18.1% three days, 33.5% four days, and 5.7% five days per week.

#### 4.1.2. Measures

**Health-oriented Leadership.** *Leaders’* and *employees’ SelfCare behavior* was assessed from the employee’s perspective using scales from the HoL inventory developed by Pundt and Felfe (2017). *Leaders*’ *SelfCare behavior* was assessed with six items covering two subfacets of SelfCare behavior: *health-promoting behavior at work* (e.g., “My supervisor ensures a healthy balance between work and personal life, e.g., by avoiding overtime, weekend, and holiday work”) and *health-risking behavior at work* (e.g., “My supervisor works more than is good for her or him”), each measured with three items. *Employees*’ *SelfCare behavior* was measured with five items on *health-promoting behavior at work* (e.g., “I also do something for my health during working hours, e.g., active breaks, healthy sitting, relaxation”) and three items on *health-risking behavior at work* (e.g., “I often find it difficult to take proper care of my health while working”). All items were rated on a 5-point Likert scale ranging from 1 = *not at all true* to 5 = *completely true*. The scales demonstrated good internal consistency, with Cronbach’s alpha values of α = 0.79 (for *leaders’ SelfCare behavior*) and α = 0.84 (for *employees’ SelfCare behavior*). The validity of the HoL scales has been established in a study introducing the instrument and providing evidence for construct validity as well as incremental validity over transformational leadership (Franke et al., 2014). The instrument has since been successfully applied across multiple samples in various occupational settings (e.g., Kaluza et al., 2021; Klug et al., 2022; Krick et al., 2024).

**Working from Home Intensity.** *Employees’ Working from Home (WfH) intensity* was measured using a single item similar to those in the studies incorporated in the meta-analysis of Gajendran and Harrison (2007). Participants answered the question “In the past four weeks during which you were working (please exclude periods of vacation, public holidays, compensatory time off, and sick leave), how often have you been working from home?” by indicating the number of days they worked from home per week using a 5-point scale (1–5 days).

**Communication Aspects.** *Communication frequency* with the direct supervisor was assessed with a single item: “In the past four weeks, how often have you been communicating with your direct supervisor?”, rated on a 5-point scale with the following response options: 1 = *very rarely (never or at most once a month)*, 2 = *every few weeks (approximately 2–3 times per month)*, 3 = *one to two times per week*, 4 = *three to four times a week,* and 5 = *every day.*

*Informal Communication* between employee and supervisor was measured using a 6-item scale developed by Lütjens and Felfe (2025), comprising two subscales: *Small Talk* (e.g., “My supervisor and I also talk about light and casual topics”; 3 items) and *Deep Talk* (e.g., “My supervisor and I also talk about personal topics, that I would not discuss with everyone, e.g., family, health”; 3 items), rated on a 5-point Likert scale ranging from 1 = *not at all true* to 5 = *completely true.* Internal consistency of the overall scale was α = 0.92.

**Control Variables.** Given that gender and age can affect health-related behaviors (e.g., Faltermaier, 1994; Schwarzer, 2004), these variables were included as controls in all our analyses. Participants reported their gender (male or female) and their age in years.

#### 4.1.3. Statistical Analysis

To predict the dependent variable (i.e., *employees*’ *SelfCare behavior*) and test H1, H3a, H5, and H6a, we conducted a Multiple Linear Regression analysis using IBM SPSS Statistics (Version 30; IBM Corp., Armonk, NY, USA). Prior to hypothesis testing, residuals of the regression model were examined for normality. Residuals were approximately normally distributed, as assessed by the Kolmogorov–Smirnov test (*p* > 0.05) and inspection of skewness and kurtosis values (|skewness| < 1, |kurtosis| < 1). Bivariate correlations were calculated using Pearson’s *r*. Following mean-centering of continuous variables, interaction terms were computed as the product of *leaders’ SelfCare behavior* with each moderator variable (i.e., *communication frequency*, *informal communication*, and *WfH intensity*). Predictors included in the model were *leaders’ SelfCare behavior*, the moderators (*communication frequency*, *informal communication,* and *WfH intensity*), the interaction terms, and the control variables (gender and age). To assess the potential risk of common method variance (CMV), three complementary analyses were conducted. First, Harman’s single-factor test indicated that the first factor accounted for 37.40% of the total variance, below the commonly used threshold of 50% (Podsakoff et al., 2003). Second, a Lindell and Whitney (2001) marker-variable approach was conducted. As no theoretically unrelated marker variable was available, the smallest observed positive correlation (*r_M* = 0.01) was used as a proxy marker variable. Given the near-zero magnitude of the marker correlation, the adjusted correlations differed only negligibly from the original values, with no changes in significance levels, suggesting that common method variance did not substantively bias the results. Third, an unmeasured latent method construct (ULMC) analysis was conducted using the lavaan package (Rosseel, 2012) in R (Version 4.5.3; R Foundation for Statistical Computing, Vienna, Austria) for the main effect variables (i.e., *leaders’* and *employees’ SelfCare behavior*). Moderator variables were excluded from the ULMC analysis as two of three moderators were assessed with single items, which precluded their inclusion as latent variables in a CFA framework. The variance of the method factor was not statistically significant (*p* = 0.338). However, the UMLC model showed improved model fit compared to baseline model and indicated changes in several factor loadings. This pattern suggests that shared variance may partly reflect measurement-related effects and/or model misspecification rather than a pure method effect. Taken together, these results do not provide consistent evidence of substantial common method variance in Study 1, although minor method-related or measurement-related effects cannot be fully ruled out.

### 4.2. Results

#### 4.2.1. Descriptive Statistics

Descriptive statistics, internal consistencies, and zero-order Pearson correlations are presented in Table 1, with correlations for Study 1 shown below the diagonal. For both leaders and employees, health-promoting and health-risking SelfCare behaviors were moderately negatively correlated. Leaders’ health-promoting, health-risking, and general SelfCare behaviors correlated moderately positively with the respective SelfCare behavior facet of the employee. Of the moderators examined, only communication frequency and informal communication were strongly positively correlated.

#### 4.2.2. Leaders’ Health-Related Role Model Effect

We postulated a positive relationship between leaders’ and employees’ SelfCare behavior in H1. A linear regression analysis was conducted to test H1, with results presented in Table 2. Controlling for age and gender, the overall model of the regression analysis revealed a positive relationship (*adj. R*^2^ = 0.27, *F*[9, 226] = 10.08, β = 0.35, *p* < 0.001), supporting H1. Higher levels of leaders’ SelfCare behavior were associated with higher levels of employees’ SelfCare behavior.

#### 4.2.3. Moderating Effects of Work Setting and Communication Aspects

**Figure 2:**
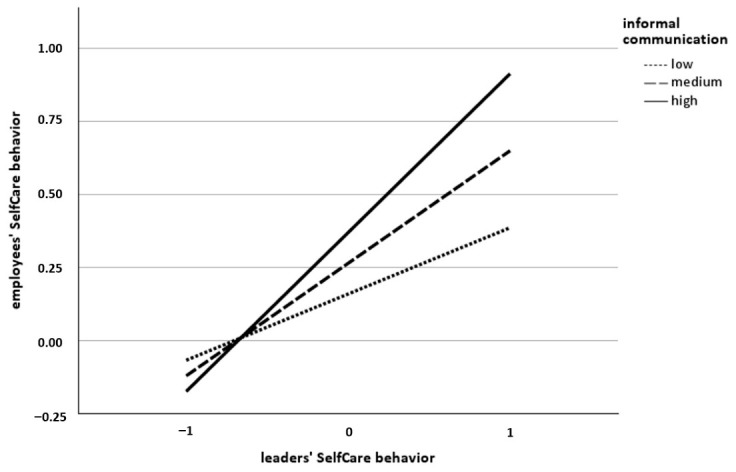
Interaction effect of leaders’ SelfCare behavior and informal communication between leader and employee (−1SD, M, 1SD) on employees’ SelfCare behavior (*N* = 227). Note. All continuous variables were z-standardized.

The postulated moderating effects of communication frequency (H3a), informal communication (H5), and WfH intensity (H6a) on the relationship between leaders’ and employees’ SelfCare behavior were tested using the same regression analysis as for H1. Contrary to our expectations, the interaction terms revealed that neither communication frequency (β = −0.04, *SE* = 0.07, *t* = −0.60, *p* = 0.547; see Table 2) nor WfH intensity (β = 0.03, *SE* = 0.05, *t* = 0.56, *p* = 0.547; see Table 2) interacted with leaders’ SelfCare behavior. Thus, H3a and H6a were not supported. However, the interaction term revealed that informal communication interacted with leaders’ SelfCare behavior (β = 0.16, *SE* = 0.07, *t* = 2.36, *p* < 0.05; see Table 2), providing support for H5. Higher levels of informal communication between leaders and employees strengthened the positive relationship between leaders’ and employees’ SelfCare behavior. The conditional effect was coeff. = 0.23 (*SE* = 0.10, *t* = 2.18, *p* < 0.05, 95% CI [0.02, 0.43]) for a low level of informal communication, coeff. = 0.39 (*SE* = 0.07, *t* = 5.84, *p* < 0.001, 95% CI [0.25, 0.52]) for a medium level of informal communication and coeff. = 0.54 (*SE* = 0.08, t = 6.52, *p* < 0.001, 95% CI [0.38, 0.70]) for a high level of informal communication. The interaction effect is visualized in Figure 2.

## 5. Study 2

### 5.1. Materials and Methods

#### 5.1.1. Participants and Procedure

Using the same approach as in Study 1, an *a priori* power analysis was conducted using G*Power (Version 3.1.9.7; Faul et al., 2009; Heinrich-Heine-Universität Düsseldorf, Düsseldorf, Germany), indicating a minimum sample size of *N* = 114 (f^2^ = 0.15, α = 0.05, 1 − β = 0.80, nine predictors). A sample of employees from a pharmaceutical company in Germany were surveyed online in late 2024. The survey was coordinated with the company’s local occupational health management and distributed to all employees working at German sites of the company via a link, although not all production employees have access to a PC and may therefore be underrepresented. The response rate was approximately 2.00%. Given that German serves as the preferred working language of the pharmaceutical company, the survey was conducted in German and sufficient language proficiency can be assumed for all participants. The survey also included leaders, as the project aimed to address a broad range of research questions. For the purposes of the present study, only data from employees without leadership responsibilities were included. Participants were informed about the voluntary nature of the study and the assured confidentiality of their data. No missing data occurred for any of the relevant study variables, as all items were set as mandatory in the online survey. A total of *N* = 199 completed the survey. Since gender was included as a control variable in the analyses and only one participant identified as non-binary, this case was excluded due to the insufficient sample size (*n* = 1) to allow for meaningful interpretation of this category. As a result, the final sample used for analysis comprised *N* = 198 participants, with 65.7% identifying as female and 34.3% as male, and an average age of 44.03 years (*SD* = 11.28). Regarding their highest educational degree, 5.6% participants stated to hold a doctoral degree, 28.3% a university degree, 37.9% a vocational or technical school training, 15.2% a higher secondary school diploma, 8.1% a secondary school diploma, and 5.0% participants held a lower secondary school diploma or another educational degree. Participants reported varying intensities of WfH: 20.7% did not work from home at all, 21.7% worked from home one day per week, 14.1% two days, 19.7% three days, 20.7% four days, and 3.0% worked full-time from home (five days per week). The majority of participants (67.7%) worked full-time.

#### 5.1.2. Measures

**Health-oriented Leadership.** *Leaders’* and *employees’ SelfCare behavior* was assessed using scales from the HoL inventory developed by Pundt and Felfe (2017) in a very similar way to Study 1. Minor adjustments were made by adding one item to each of the subscales measuring *leaders’ health-promoting behavior, employees’ health-promoting behavior,* and *employees’ health-risking behavior at work.* Consequently, the scale for *leaders*’ *SelfCare behavior* comprised 7 instead of 6 items (α = 0.87), and the scale for *employees*’ *SelfCare behavior* comprised 9 instead of 7 items (α = 0.88) in Study 2.

**Disclosure.** *Leaders’ disclosure of their mental health problems or psychological strain to their employees* and *employees’ disclosure of their mental health problems or psychological strain to their leaders* were measured from the employee’s perspective, using scales developed by Pischel and Felfe (2023), with wording adapted to the respective perspective (leader or employee). Before completing the items, participants were instructed to indicate how they and their leader currently handle, or would handle, mental health problems or psychological strain at work. These concepts were broadly defined to encompass all work-related factors that may affect employees or leaders (e.g., excessive workload, interpersonal conflicts, and lack of support). Participants who were not currently experiencing such problems or strain were explicitly asked to imagine a corresponding situation and indicate how they would behave in their current work context. Accordingly, the measure captured employees’ general openness toward their leader regarding mental health-related concerns, as well as their perceptions of their leader’s openness toward them, incorporating both actual and hypothetical disclosure behavior. The scales for *leaders’ disclosure* (e.g., “My leader keeps mental health problems or psychological strain to themselves and does not share it with me or would keep it to themselves and would not share it with me” (reversed coded); α = 0.78) and *employees’ disclosure* (e.g., “I confide my mental health problems and psychological strain in my supervisor, or would confide in them”; α = 0.92) each consisted of four items, rated on a 5-point Likert scale ranging from 1 = *not at all true* to 5 = *completely true*.

**Working from Home Intensity.** The measurement of *employees’ Working from Home (WfH) intensity* was conducted very similarly to Study 1, with a new response option, “never”, added to the scale, creating a 6-point scale instead of the 5-point scale employed in Study 1.

**Communication Aspects.** *Communication frequency* with the supervisor was measured in the same way as in Study 1. *Communication barriers* encountered by employees with their leaders were assessed using an adapted scale based on the subscale “poor communication” of the ICT demands scale by Day et al. (2012), consisting of three items (e.g., “Misunderstandings occur during communication or coordination with my leader”; α = 0.90) and assessed on a 5-point Likert-scale from 1 = *never* to 5 = *almost always*.

**Control Variables.** Following the same reasoning and measurement as in Study 1, gender and age were included as control variables to account for their effects in the analyses. Participants reported their age in years and gender (male, female, or non-binary).

#### 5.1.3. Statistical Analysis

Using the same approach as in Study 1, two Multiple Linear Regression analyses were performed using IBM SPSS Statistics (Version 30; IBM Corp., Armonk, NY, USA). Prior to conducting the main analyses, the residuals of both regression models were checked for normality. Results indicated that residuals were approximately normally distributed across both models, as supported by the Kolmogorov–Smirnov test (*p* > 0.05) and skewness and kurtosis values within acceptable ranges (|skewness| < 1, |kurtosis| < 1). Pearson’s *r* was used to calculate bivariate correlations between all study variables. After mean-centering of the continuous variables, interaction terms were created by multiplying (1) *leaders’ SelfCare behavior* with each moderator variable (i.e., *communication frequency*, *communication barriers, and WfH intensity*) and (2) *leaders’ disclosure* with each of the moderators. To predict the dependent variable, *employees*’ *SelfCare behavior,* and test H1, H3a, H4a, and H6a, a Multiple Linear Regression analysis was conducted, including *leaders’ SelfCare behavior*, the moderators, and the interaction terms (products of the moderators and *leaders’ SelfCare behavior*) as predictors. Additionally, to test H2, H3b, H4b, and H6b and predict the dependent variable, *employees*’ *disclosure,* we included *leaders’ disclosure*, the moderators, and the interaction terms (products of the moderators and *leaders’ disclosure*) as predictors in the second Multiple Linear Regression analysis. Gender and age were added as control variables in both analyses. To assess the potential risk of common method variance (CMV), the same three complementary analyses as in Study 1 were conducted. Harman’s single-factor test indicated that the first factor accounted for 35.20% of the total variance, below the recommended threshold of 50% (Podsakoff et al., 2003). Second, a Lindell and Whitney (2001) marker-variable correction was conducted. Similar to Study 1, the smallest observed positive correlation (*r_M* = 0.01) was used as a proxy marker variable, as no theoretically unrelated marker variable was available. Adjusted correlations were computed, which were virtually identical to the original correlations, and no changes in statistical significance were observed. Third, an unmeasured latent method construct (ULMC) analysis was conducted using the lavaan package (Rosseel, 2012) in R (Version 4.5.3; R Foundation for Statistical Computing, Vienna, Austria) for the main effect variables (i.e., *leaders’* and *employees’ SelfCare behavior* and *leaders’* and *employees’ disclosure*). As in Study 1, moderator variables were excluded from the ULMC analysis due to the single-item measurement of two of three moderators. The variance of the method factor was statistically significant (*p* < 0.05). Additionally, the ULMC model showed improved fit compared to the baseline model. However, the method loadings showed an inconsistent pattern across indicators and constructs, suggesting that the method factor does not reflect a uniform systematic response bias. Overall, these findings indicate a small to moderate method effect in Study 2, but no evidence that this effect substantially biases the substantive relationships.

### 5.2. Results

#### 5.2.1. Descriptive Statistics

Descriptive statistics, internal consistencies, and zero-order Pearson correlations of the variables are reported in Table 1 (correlations for Study 2 are presented above the diagonal). Moderate to strong negative intercorrelations were found between the two subfacets, health-promoting and health-risking SelfCare behaviors, for both leaders and employees. Leaders’ health-promoting, health-risking, and general SelfCare behavior correlated weakly to moderately positively with the respective SelfCare behavior facet of their employees. Leaders’ disclosure correlated moderately positively with employees’ disclosure. While WfH intensity did not correlate with communication barriers, it showed a weak negative correlation with communication frequency. Communication frequency and communication barriers showed a weak negative intercorrelation.

#### 5.2.2. Leaders’ Health-Related Role Model Effect

Two Multiple Linear Regression analyses were conducted to test the hypotheses regarding leaders’ health-related role model effect in terms of SelfCare behavior (H1) and the disclosure of mental health problems (H2). The results of all regression analyses are presented in Table 2.

**Leaders’ SelfCare role model effect.** Controlling for age and gender, the regression analysis model revealed a positive relationship between leaders’ and employees’ SelfCare behavior (*adj. R*^2^ = 0.12, *F*[9, 197] = 4.01, β = 0.30, *p* < 0.001), supporting H1. Higher levels of leaders’ SelfCare behavior were associated with higher levels of employees’ SelfCare behavior.

**Leaders’ disclosure role model effect.** Regarding disclosure, the regression analysis revealed a positive relationship between leaders’ and employees’ disclosure of mental health problems or psychological strain (*adj. R*^2^ = 0.39, *F*[9, 197] = 14.97, β = 0.35, *p* < 0.001), controlled for age and gender, thus supporting H2. Higher levels of leaders’ disclosure were associated with higher levels of employees’ disclosure.

#### 5.2.3. Moderating Effects of Work Setting and Communication Aspects

**Figure 3:**
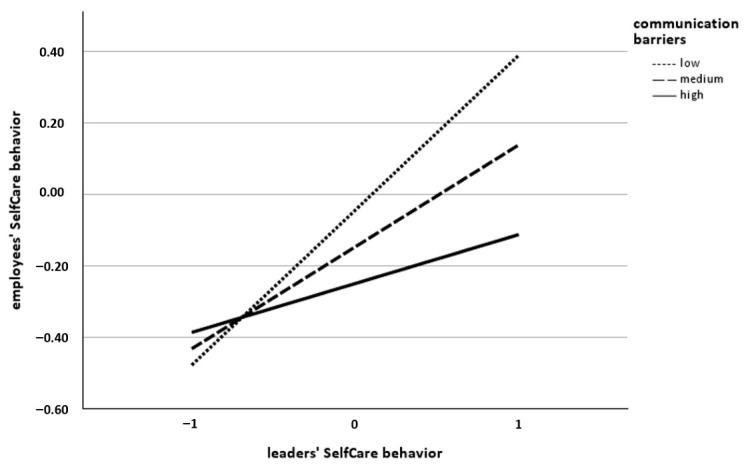
Interaction effect of leaders’ SelfCare behavior and communication barriers between leader and employee (−1SD, M, 1SD) on employees’ SelfCare behavior (*N* = 198). Note. All continuous variables were z-standardized.

**Leaders’ SelfCare role model effect.** The postulated moderating effects of communication frequency (H3a), communication barriers (H4a), and WfH intensity (H6a) on the relationship between leaders’ and employees’ SelfCare behavior were examined using the same regression analytic approach as for H1. Contrary to our hypotheses, the interaction terms revealed that neither communication frequency (β = 0.00, *SE* = 0.07, *t* = 0.20; *p* = 0.984; see Table 2) nor WfH intensity (β = −0.04, *SE* = 0.05, *t* = 0.60, *p* = 0.551; see Table 2) moderated the relationship between leaders’ and employees’ SelfCare behavior, providing no support for H3a or H6a. However, for communication barriers, the interaction term revealed a significant interaction with leaders’ SelfCare behavior (β = −0.17, *SE* = 0.07, *t* = -2.20, *p* < 0.05; see Table 2), supporting H4a. Higher levels of communication barriers between leaders and employees weakened the relationship between leaders’ and employees’ SelfCare behavior. The conditional effect was coeff. = 0.43 (*SE* = 0.10, *t* = 4.15, *p* < 0.001, 95% CI [0.23, 0.64]) for a low level of communication barriers, coeff. = 0.29 (*SE* = 0.07, *t* = 3.99, *p* < 0.001, 95% CI [0.15, 0.43]) for a medium level of communication barriers and coeff. = 0.14 (*SE* = 0.09, *t* = 1.50, *p* = 0.134, 95% CI [−0.04, 0.32]) for a high level of communication barriers. The interaction effect is displayed in Figure 3.

**Figure 4:**
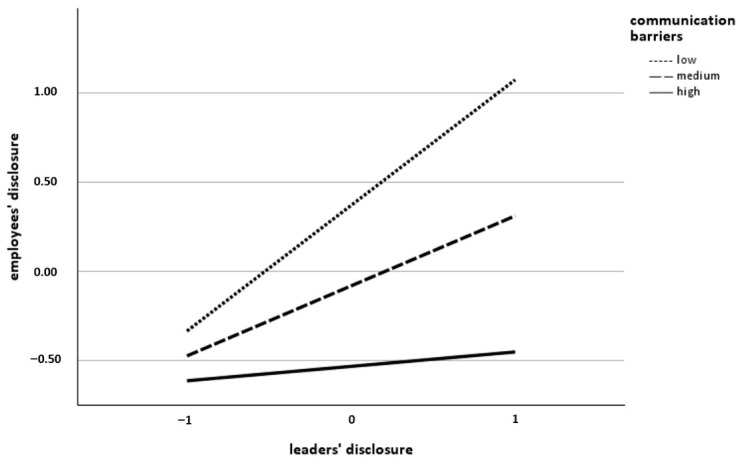
Interaction effect of leaders’ disclosure and communication barriers between leader and employee (−1SD, M, 1SD) on employees’ disclosure (*N* = 198). Note. All continuous variables were z-standardized.

**Leaders’ disclosure role model effect.** The hypothesized moderating effects of communication frequency (H3b), communication barriers (H4b), and WfH intensity (H6b) on the relationship between leaders’ and employees’ disclosure of mental health problems or psychological strain were tested using the same regression analysis applied to examine H2. Contrary to our expectations, the interaction terms indicated that leaders’ disclosure did not interact with either communication frequency (β = −0.08, *SE* = 0.07, *t* = −1.30, *p* = 0.197; see Table 2) or WfH intensity (β = 0.05, *SE* = 0.05, *t* = 0.80, *p* = 0.427; see Table 2). Accordingly, H3b and H6b were not supported. However, the interaction term for communication barriers was significant (β = −0.27, *SE* = 0.07, *t* = −4.18, *p* < 0.001; see Table 2), providing support for H4b. Higher levels of communication barriers between leaders and employees attenuated the positive relationship between leaders’ and employees’ disclosure. The conditional effect was coeff. = 0.70 (*SE* = 0.11, *t* = 6.57, *p* < 0.001, 95% CI [0.49, 0.91]) for a low level of communication barriers, coeff. = 0.39 (*SE* = 0.07, *t* = 5.45, *p* < 0.001, 95% CI [0.25, 0.35]) for a medium level of communication barriers and coeff. = 0.08 (*SE* = 0.10, *t* = 0.81, *p* = 0.420, 95% CI [−0.12, 0.28]) for a high level of communication barriers. The interaction effect is illustrated in Figure 4.

## 6. Discussion

The present studies examined leaders’ role-modeling in terms of SelfCare and, specifically, the disclosure of mental health problems as a sensitive form thereof. Based on the HoL Model (Franke et al., 2014), we investigated whether the role-modeling pattern observed for general SelfCare also applies to this more sensitive behavior. We further examined the contextual and interpersonal conditions under which these role-modeling patterns occur. Our findings provide empirical support for the hypothesized positive relationships between leaders’ and employees’ health-related behaviors. They also offer novel insights into the mechanisms of social learning, as conceptualized by Bandura (1969, 1971), in organizational health contexts, revealing specific communication aspects as relevant boundary conditions. The findings are discussed below.

### 6.1. Leaders’ as Role Models

Consistent with prior research (Fröhlich et al., 2025; Klug et al., 2019), Study 1 demonstrated that leaders’ SelfCare behaviors—such as taking regular breaks and seeking support when needed—were positively associated with employees’ own SelfCare behaviors. These findings provide further support for the role-modeling mechanism outlined in the HoL Model (Franke et al., 2014), highlighting that leaders’ personal health practices are related to employees’ health behaviors. Study 2 extended these insights by showing that leaders’ disclosure of mental health problems was similarly associated with employees disclosing their own mental health problems to their leaders. This aligns with evidence suggesting that disclosure is a reciprocal social process (Cozby, 1973; Martinez et al., 2014) and supports prior findings on leaders’ role modeling of health-related behaviors beyond general SelfCare, including work–life segmentation (A. R. Koch & Binnewies, 2015) and presenteeism (Dietz et al., 2020). Our results provide initial indications that employees’ decisions to disclose psychological strain—a complex and often challenging choice (Brohan et al., 2012)—may be associated with leaders’ behavior. This extends prior research on leaders’ influence on employees’ disclosure decisions, showing that, beyond the positive effects of leaders’ StaffCare (Pischel & Felfe, 2023), leaders’ own disclosure may serve as an additional role-modeling mechanism shaping employees’ health-related behavior.

By modeling SelfCare and disclosure behaviors, leaders can establish norms of acceptable health-related practices, encouraging employees not only to engage in SelfCare but also to communicate openly about mental health problems. This aligns with the already established potential of leaders to contribute to psychologically safe work environments (e.g., Clarke et al., 2025) and to a culture where mental health is openly acknowledged and supported.

### 6.2. Moderating Factors of the Role-Modeling Pattern

We postulated interpersonal boundary conditions (i.e., communication dynamics) and a contextual factor (i.e., WfH intensity) to moderate leaders’ role-modeling pattern regarding SelfCare behavior and the disclosure of mental health problems. More specifically, based on the Job Demands-Resources model (Bakker & Demerouti, 2007, 2017; Demerouti et al., 2001), we expected frequent communication to be a job resource for employees and therefore to be associated with a stronger leaders’ SelfCare and disclosure role modeling. Conversely, we expected high WfH intensity and communication barriers to represent job demands for employees in this context. Moreover, we considered informal communication to be a particularly supportive resource that specifically enhances leaders’ SelfCare role-modeling pattern. While previous research has shown that communication frequency, in addition to communication barriers and informal communication, positively contributes to other leadership aspects such as leaders’ StaffCare (Bruhn et al., 2025), our findings suggest that, regarding the role modeling of health-related behaviors, it is the quality and content of communication rather than its frequency that are decisive. This interpretation aligns with evidence from other organizational contexts, where communication quality was found to matter more than frequency for fostering organizational trust (van Zoonen et al., 2024) and reducing burnout (Shockley et al., 2021). Interestingly, contrary to our expectations, the frequency of communication between leaders and their employees was not associated with a stronger role-modeling pattern for either SelfCare or disclosure. Several possible explanations may account for this. First, mere exposure to the leader through frequent communication might not be sufficient for employees to perceive and emulate their leaders’ health behaviors. Regular exchanges do not necessarily imply meaningful or high-quality interactions; they may be primarily task-oriented or superficial, thus failing to foster openness, trust, or relational closeness, which are important elements of social learning (Bandura, 1969). Even with frequent communication, health-related topics such as SelfCare or mental health disclosure may simply not be discussed, leaving few opportunities for modeling to occur. Communication frequency may be mostly shaped by the nature of the tasks and employees who communicate frequently with their leaders may do so primarily for task coordination purposes. This would mean that the content of these exchanges remains work-focused rather than health-related. Thus, high communication frequency may in this regard reflect task interdependence rather than relational closeness and may therefore not create the conditions necessary for health-related role modeling to occur. Second, contrary to our assumptions, more frequent communication might not necessarily enhance the visibility of leaders’ health-related behaviors. Continuous streams of information can lead to reduced attention to specific cues, such as SelfCare or mental health disclosure, due to information overload (Arnold et al., 2023; Graf & Antoni, 2023). According to the Social Learning Theory (Bandura, 1969), attention and perceived relevance are prerequisites for observational learning. Thus, while frequent communication provides more opportunities for interaction, it does not appear to automatically increase employees’ capacity to observe and emulate their leaders’ health behaviors. Beyond these theoretical explanations, it should also be acknowledged that methodological factors may have contributed to the non-significant moderation, including the single-item operationalization of communication frequency, restricted variability in responses, suggesting a ceiling effect, and sample-specific characteristics. These aspects are discussed in more detail in the Limitations section.

On the contrary, as expected, communication quality and content appear to be decisive for the leaders’ health-related role model effect. Specifically, in Study 2, communication barriers—considered an aspect of communication quality (Marlow et al., 2017)—were associated with a weakened role model effect of SelfCare and disclosure. We assume that ambiguous or unclear communication, as well as potential misunderstandings, may hinder social learning mechanisms (Bandura, 1969, 1971, 1986) by reducing trust and the perceived quality of leader–employee relationships. Accordingly, communication barriers are likely to limit the extent to which leaders are perceived as credible and authentic role models (Kamal Bahrain et al., 2023; Tautz et al., 2024). More generally, this aligns with Bruhn et al. (2025), who found that perceived communication barriers negatively affect leaders’ StaffCare, indicating that such obstacles can undermine leaders’ health-oriented influence on employees.

Conversely, as expected, informal communication was associated with a stronger relationship between leaders’ and employees’ SelfCare behaviors in Study 1. We attribute this effect to several mechanisms: Informal interactions may increase the salience of leaders’ behavior, contribute to relational closeness, and strengthen perceptions of credibility and authenticity. Specifically, informal interactions may promote trust and identification with the leader, thereby enhancing employees’ perception of the leader as a credible role model (Bandura, 1969; Kelman, 1958). In addition, informal communication provides opportunities to exchange personal or health-related information, which can make SelfCare behaviors more visible and concrete, ultimately increasing the likelihood of behavioral imitation (Bandura, 1969, 1971; Fay & Kline, 2011). More generally, these mechanisms are consistent with prior findings showing that increased informal communication enhances employees’ perceptions of Transformational Leadership (Lütjens & Felfe, 2025) and StaffCare (Bruhn et al., 2025).

Contrary to theoretical expectations derived from Media Richness Theory (Daft & Lengel, 1986) and prior assumptions and findings on virtual leadership (Tautz et al., 2022; Yue et al., 2021), our results indicate that WfH intensity did not hinder employees’ ability to observe or imitate leaders’ health-related behaviors. While these theories suggest that reduced F2F contact and the absence of nonverbal cues could diminish perceptions of authenticity or make the interpretation of ambiguous behaviors more difficult, our findings imply that employees may still successfully perceive leaders as role models. This is somehow broadly consistent with a finding of Lundqvist et al. (2022), who underscore that the leadership of employees’ manager matters for employee well-being regardless of whether they work on-site or from home. One possible explanation for our findings may be that digital communication channels—such as video calls, chat, or e-mail—can convey important cues about leaders’ health behavior even in the absence of frequent F2F contact. For example, leaders may intentionally highlight their SelfCare routines in team chats or share personal experiences related to mental health during video meetings, thereby maintaining visibility and signaling authenticity. Similar to our findings on communication frequency, it appears that the intensity of WfH does not automatically determine leaders’ role model effects. Instead, as with communication, it seems that the quality and content of interactions—for example, whether communication is clear, informative, and informal—may be more decisive for employees’ observation and emulation of health-related behaviors. Our findings suggest that even high WfH intensity, which reduces F2F contact (e.g., Lal et al., 2021), does not necessarily weaken leaders’ role model effect. In remote or hybrid work settings, it may be particularly important how and about what leaders communicate with their employees, as these aspects likely determine whether they are perceived as authentic role models for SelfCare and the disclosure of mental health issues. In addition to these theoretical considerations, methodological factors such as the single-item measurement of WfH intensity may have limited the ability to detect interaction effects. These points are addressed in greater detail in the Limitations section.

### 6.3. Theoretical and Practical Implications

Our studies contribute to theory in several ways. First, they provide empirical support for the extension of leaders’ role model effect within the HoL Model (Franke et al., 2014) to more sensitive forms of health-related behavior, namely the disclosure of mental health problems or psychological strain. This also complements existing models of disclosure (e.g., Jones & King, 2014; Ragins, 2008; Toth & Dewa, 2014) by identifying leaders’ self-disclosure to their employees as a novel antecedent for employees’ disclosure of mental health problems. Second, the findings offer further evidence for the Social Learning Theory (Bandura, 1969, 1971) and the Social Cognitive Theory (Bandura, 1986) in organizational settings, demonstrating that leaders’ own health behaviors serve as salient models for employees. Third, by examining contextual and interpersonal moderators, our research responds to calls for more nuanced and context-sensitive leadership studies (Johns, 2024; De Vries et al., 2010). We extend the work of Fröhlich et al. (2025) by advancing the HoL Model (Franke et al., 2014) to include interpersonal boundary conditions that are associated with whether employees imitate leaders’ health-related behaviors—namely, communication dynamics. Specifically, we integrate communication barriers between leaders and employees as a hindering factor for the role-modeling pattern, while incorporating informal communication as a facilitating condition.

Building on these findings, our studies contributes to ongoing discussions about which challenges or resources predominate when working from home (e.g., Babapour Chafi et al., 2022; Hill et al., 2024). In particular, Eisenberg et al. (2019) and Tautz et al. (2022) suggested that leaders in remote work contexts might be perceived as less authentic by employees, potentially diminishing their role model effect. Our results, however, indicate that regarding health-related role modeling, leaders’ role-modeling pattern is not systematically associated with simply the extent to which employees work from home. This aligns with a growing body of research suggesting that the effects of remote work depend on how work is designed and structured, rather than on the work mode itself (e.g., Hackney et al., 2022). In this regard, our studies also contributes to the debate raised by Krick and Felfe (2022) on whether the role model effect regarding leaders’ health-related behaviors remains effective in digital or hybrid work settings. Our findings suggest that it can, provided that appropriate communication practices are in place.

Furthermore, our findings contribute to the growing literature on informal communication that underscores the importance of informal communication for leadership perception and effectiveness in terms of Transformational Leadership (Lütjens & Felfe, 2025; Tautz et al., 2023) and HoL (Bruhn et al., 2025). Our first study complements and extends this by showing that informal interactions are positively associated with leaders’ role-modeling of health-related behaviors, highlighting that spontaneous, non-work-related exchanges may be crucial for leaders’ health-related role-modeling pattern.

Finally, our studies integrate the HoL Model (Franke et al., 2014) with the JD-R framework (Bakker & Demerouti, 2007, 2017; Demerouti et al., 2001), showing that communication barriers represent a job demand that can hinder employees’ emulation of leaders’ health-related behaviors, whereas informal communication functions as a job resource that facilitates health-related role modeling.

From a practical perspective, the findings underline the importance of enhancing leaders’ awareness of their role-model function for employee health. Our cross-sectional findings provide further evidence for the role of leaders’ SelfCare behavior (e.g., taking regular breaks, avoiding overtime) as a catalyst for employees’ own health-related actions, complementing StaffCare. Moreover, they offer preliminary support for the notion that leaders’ disclosure of psychological strain may similarly serve as a powerful role model behavior. Leadership development initiatives—such as the “Go-FüKo” training, which trains leaders’ HoL competencies (Krick & Felfe, 2024)—should therefore sensitize leaders to the positive impact of modeling health-related behavior and train them to make these behaviors visible across different work settings. Although the cross-sectional nature of our findings calls for caution in deriving practical recommendations, the results suggest several tentative implications for organizational practice. Organizations should create supportive conditions that enable leaders to authentically engage in SelfCare and, when appropriate, disclose their own psychological strain to their employees as a positive example. Reducing barriers that inhibit such behaviors may be essential to ensure that leaders feel comfortable and confident in acting as positive health-related role models, thereby potentially encouraging employees to open up about their own struggles and seek support when needed. Second, our findings suggest that communication may constitute a crucial condition for the leaders’ health-related role-modeling pattern. Communication barriers between leaders and employees were associated with weaker relationships between leaders’ and employees’ SelfCare and disclosure behaviors. On the contrary, informal communication—comprising Small Talk and deeper, meaningful work-related conversations (Deep Talk)—was associated with a stronger relationship between leaders’ and employees’ SelfCare behavior. In line with Bravo-Duarte et al. (2025), who emphasize that leaders of remote teams require specific competencies to promote employee well-being, our results indicate that communication effectiveness may be a key competence in this regard. Based on these preliminary findings, leadership development programs could therefore strengthen communication skills and include targeted training on how to reduce communication barriers and purposefully integrate informal exchanges with employees into everyday work routines. Such training may focus on message clarity and structured informal check-ins, while also teaching techniques such as closed-loop communication (Marlow et al., 2017) to prevent misunderstandings and, consequently, potentially strengthen leaders’ effectiveness as health-related role models in both remote and co-located settings. However, these recommendations should be treated as tentative until replicated in longitudinal or experimental designs.

### 6.4. Strengths, Limitations, and Future Research

Although the presented studies have several notable strengths, they are not without limitations. The research offers valuable empirical insights by investigating leaders’ health-related role-modeling patterns in the context of SelfCare and the disclosure of mental health problems across two distinct organizational settings—a pharmaceutical company and a public organization. The use of two independent samples enhances the robustness and external validity of the findings. Moreover, by capturing health-related role modeling processes in modern hybrid and remote work contexts, the studies contribute timely evidence on how communication dynamics, namely informal communication and communication barriers, are linked to the transmission of health-related behaviors between leaders and employees. These strengths increase confidence in the generalizability and practical relevance of the findings.

Nevertheless, several limitations must be acknowledged, which also provide promising directions for future research. First, the response rates in both studies were low, particularly in Study 2 (2.0%), likely due to survey fatigue, as employees had recently participated in an unrelated survey. This may limit the representativeness of the samples and should therefore be taken into account when interpreting the findings. However, as the present studies focus on relational rather than distributional questions, the risk of bias resulting from the low response rate is comparatively limited (Rindfuss et al., 2015).

Second, since all variables were measured at the same time point, common method bias cannot be excluded. Specifically, since participants rated both their own health behavior and their leaders’ health behavior, shared method variance may partly explain the observed associations, as ratings from a single source may inflate correlations between variables. For instance, employees who perceive their leaders as engaging in SelfCare or disclosure behavior may be more likely to report their own corresponding behaviors positively, independent of the true relationship between leaders’ and employees’ behaviors. While both Harman’s single-factor test and Lindell and Whitney (2001) marker-variable approach did not indicate substantial concerns for the present studies, the ULMC analysis suggests that limited and non-systematic method-related variance is present across the two studies. In Study 2, the ULMC results suggest the presence of a small to moderate method effect, which should be considered when interpreting the findings of this study. However, this effect does not appear to substantially alter the substantive conclusions when considered alongside the marker-variable results. Future research should ideally employ longitudinal or multi-source designs to more rigorously address this concern, for example, by assessing the dependent variable at a separate time point. Furthermore, the cross-sectional design in both studies limits conclusions regarding causality and the direction of effects. Although leaders’ SelfCare and disclosure behaviors were associated with employees’ corresponding behaviors, the data do not allow for determining whether leaders’ behaviors cause changes in employees’ behaviors or whether reciprocal effects exist. Nonetheless, as the interpretation of the findings is grounded in the Social Learning Theory (Bandura, 1969) and the HoL Model (Franke et al., 2014), as well as it aligns with longitudinal evidence showing leader–employee transmission effects for SelfCare (Fröhlich et al., 2025) and presenteeism (Dietz et al., 2020), we cautiously infer a potential role model effect of leaders, while acknowledging that the cross-sectional design does not allow for causal conclusions. As Spector (2019) notes, cross-sectional designs are a valuable and legitimate tool in the early stages of research, particularly for establishing whether theoretically expected associations exist in the data and for generating hypotheses for future longitudinal or experimental studies. The present studies serve precisely this purpose by providing initial cross-sectional evidence for theoretically expected associations that have not previously been examined empirically and laying the groundwork for more rigorous causal designs. Future studies should employ longitudinal or experimental designs to establish causal direction more clearly, for example, by measuring leaders’ and employees’ behavior at different time points or by manipulating leaders’ behaviors and measuring their effects on employees’ subsequent behaviors.

Third, all variables were assessed through employee self-reports, which introduces the risk of common source bias. This approach was chosen because, according to Social Learning Theory (Bandura, 1969), only behavior that is sufficiently visible, observable, and actually perceived by employees can be imitated, meaning that leaders’ self-reported behaviors may not necessarily reflect what employees actually perceive. Nevertheless, future studies should incorporate both leaders’ and employees’ perspectives to examine potential discrepancies between leaders’ intended behaviors and employees’ perceptions, as discrepancies between these two perspectives may themselves constitute an important boundary condition of the role model effect. Building on this, two promising directions for future research emerge: First, comparing leaders’ self-assessments with employees’ perceptions of their health-related behaviors would shed light on the extent to which role modeling is contingent on the accuracy of leaders’ self-awareness. Second, drawing on Felfe and Heinitz (2010), who showed that team consensus in ratings of transformational leadership indicates consistent leadership behavior and moderates organizational outcomes, future research could examine the degree of consensus within teams regarding leaders’ SelfCare and disclosure behaviors, with high consensus potentially indicating consistent role modeling and low consensus suggesting that leaders’ behaviors are perceived inconsistently across team members. Prior to examining such questions, however, it was aimed to first establish whether employees’ perceptions of leaders’ health-related behaviors are associated with their own behaviors, as employees’ perceptions represent the most fundamental prerequisite for observational learning according to Social Learning Theory (Bandura, 1969).

Fourth, although the current research examined both interpersonal and contextual moderators, future studies should extend the analysis to team-level factors. For example, PeerCare—the extent to which team members value their colleagues’ health, recognize health warning signs in them, and actively take care of their colleagues’ health (Gosch et al., 2023)—may amplify or buffer the effects of leaders’ health-related role modeling. Furthermore, the present studies did not control for potentially relevant third variables that may have influenced both leaders’ health-related behaviors and employees’ tendency to emulate them. Drawing on evidence that psychological safety climate shapes employees’ mental health (e.g., Amoadu et al., 2024) and well-being (e.g., Shee et al., 2026) a positive psychological safety climate may also independently promote employees’ willingness to engage in SelfCare and to disclose mental health problems, regardless of leaders’ behaviors. Moreover, psychological safety may also moderate the role model effect itself. In climates where openness and vulnerability are generally valued, employees may be more attentive to and more willing to emulate leaders’ health-related behaviors. Conversely, in psychologically unsafe climates, even leaders who openly engage in SelfCare or disclose mental health problems may not inspire imitation, as employees may fear negative consequences. Another relevant variable may be the leadership style: For example, transformational leaders, who are characterized by idealized influence and role modeling (Bass & Avolio, 1994a), may be particularly effective health-related role models compared to other leadership styles. Conversely, transactional leaders communicate less openly about health-related topics, potentially reducing the visibility of their SelfCare behaviors. Future research should therefore examine both psychological safety climate and leadership style as potential boundary conditions and confounding variables of leaders’ health-related role modeling.

Fifth, in the presented studies, WfH intensity and communication frequency were examined as moderators of leaders’ role-modeling pattern, reflecting the realities of modern hybrid and remote work structures. However, neither of these two moderation effects was significant. From a theoretical perspective, regarding WfH intensity, simply measuring the intensity of WfH days may not capture the factors that influence role model processes. Examining qualitative aspects, such as the design, quality, and social nature of virtual collaboration, would provide a deeper understanding of these boundary conditions and aligns with research indicating that the effects of remote work depend more on how work is structured than on the work mode itself (e.g., Hackney et al., 2022). For example, Klebe et al. (2023) found that while HoL in terms of StaffCare positively affects employees when working from home, its effectiveness may be impaired by technical issues such as Information and Communication Technology (ICT) hassles, suggesting that specific conditions during WfH, such as the quality of digital infrastructure, may constitute relevant boundary conditions of the role model effect worth examining in future research. Considering these factors may clarify under which conditions employees can effectively observe and learn from their leaders’ health-related behaviors in remote environments, in addition to the communication aspects that were found to be decisive in our studies. Beyond these theoretical considerations, several methodological factors may have additionally contributed to the non-significant findings. Both WfH intensity and communication frequency were each assessed with single items, which may not have fully captured their complexity. The operationalization of communication frequency, in particular, may have been too coarse to detect meaningful differences, potentially failing to capture the nuanced differences in interaction patterns relevant for health-related role modeling. While single-item measures can be acceptable and valid (Allen et al., 2022; Gajendran & Harrison, 2007), more fine-grained multi-item measures would allow for a more precise assessment of both constructs. Moreover, inspection of the response distribution for communication frequency revealed restricted variability, with the majority of participants reporting frequent communication. This ceiling effect may have substantially limited the ability to detect differential moderation effects across levels of communication frequency. Additionally, sample-specific characteristics may have contributed to the non-significant moderation effect of communication frequency. Both samples were drawn from rather formally structured organizations, potentially incorporating rather task-oriented communication. In such contexts, even frequent communication may rarely touch on health-related topics, potentially limiting the opportunity for communication frequency to unfold its expected moderating role. In organizational contexts characterized by less formal structures and more relational communication patterns, communication frequency may be a more meaningful boundary condition of leaders’ health-related role modeling. Future research should employ validated multi-item measures with more fine-grained response scales for both constructs and examine these moderators in more diverse organizational contexts characterized by less formal structures, to allow for a more precise and reliable assessment of their role as boundary conditions of leaders’ health-related role modeling.

Further research should explore the motivational and cognitive processes underlying employees’ decisions to imitate or not imitate their leaders’ health-related behaviors. Qualitative or mixed-method approaches, such as interviews, could help uncover employees’ reasoning and contextual influences on imitation processes.

Moreover, situational factors such as crises or times of collective strain may alter the relevance of leaders as health role models. Previous research (e.g., Klebe et al., 2021) suggests that leaders’ StaffCare becomes particularly important in times of crisis. Future studies should examine whether such situations also increase leaders’ health-related role model effect or whether crises shift attention away from leaders’ behaviors, thereby potentially weakening the modeling effect.

Finally, future research should examine the barriers that prevent leaders from engaging in health-related disclosure or SelfCare, which may include concerns about losing authority, perceived stigma, or organizational expectations. A better understanding of these barriers could help explore the conditions under which leaders can demonstrate positive role model behaviors in the first place.

## 7. Conclusions

In sum, our findings demonstrate that leaders’ SelfCare and disclosure of mental health problems or psychological strain are positively associated with employees’ respective health behaviors, with the role-modeling pattern contingent on communication quality and content. While communication barriers were associated with weaker leaders’ health-related role-modeling patterns, informal communication between leaders and employees was associated with a stronger SelfCare role-modeling pattern. These results suggest the relevance of leaders’ role modeling behavior for health-supportive workplaces and highlight the importance of reducing communication barriers and enhancing informal communication as factors associated with employees’ adoption of leaders’ health-related behaviors. Our studies extend theoretical frameworks on health-oriented leadership (Franke et al., 2014), social learning (Bandura, 1969), and disclosure (e.g., Ragins, 2008; Toth & Dewa, 2014) and offer practical insights for leaders and organizations aiming to promote employees’ health behaviors through leadership practices.

## Figures and Tables

**Table 1:** Descriptive statistics and zero-order Pearson correlations of all study variables; correlations for study 1 are below diagonal, correlations for study 2 above.

	Study 1		Study 2		1	2	3	4	5	6	7	8	9	10	11	12	13	14
	*M (SD)*	α	*M (SD)*	α														
**Control variables**																		
1 Gender ^a^	-	-	-	-	-	−0.11	0.06	0.09	−0.01	**0.16**	**0.18**	−0.09	0.06	0.03	**0.19**	0.06	−0.06	-
2 Age	44.07 (10.65)	-	44.03 (11.28)	-	**−0.17**	-	−0.07	−0.05	0.07	0.06	0.11	0.04	0.12	**0.17**	**0.22**	−0.06	−0.01	-
**Leader SelfCare Behavior**																		
3 General	3.36 (0.66)	0.79	3.12 (0.79)	0.87	−0.03	0.01	-	**0.89**	**−0.82**	**0.30**	**0.24**	**−0.31**	**0.21**	**0.26**	−0.03	0.05	**−0.33**	-
4 Promotion	3.47 (0.74)	0.91	3.24 (0.90)	0.87	−0.02	−0.02	**0.77**	-	**−0.48**	**0.27**	**0.22**	**−0.27**	**0.25**	**0.35**	0.00	0.13	**−0.46**	-
5 Risk	2.75 (0.89)	0.81	3.04 (0.95)	0.88	0.03	−0.03	**−0.85**	**−0.32**	-	**−0.24**	**−0.19**	**0.27**	−0.09	−0.06	0.04	0.07	0.07	-
**Employee SelfCare Behavior**																		
6 General	3.49 (0.73)	0.84	3.24 (0.76)	0.88	−0.06	0.04	**0.44**	**0.43**	**−0.30**	-	**0.94**	**−0.82**	**0.21**	**0.33**	0.08	0.11	**−0.21**	-
7 Promotion	3.65 (0.79)	0.78	3.36 (0.79)	0.86	0.02	0.02	**0.34**	**0.41**	**−0.17**	**0.94**	-	**−0.57**	**0.22**	**0.31**	0.09	0.09	**−0.15**	-
8 Risk	2.92 (0.95)	0.84	3.00 (0.98)	0.86	**0.20**	−0.05	**−0.48**	**−0.30**	**0.46**	**−0.72**	**−0.45**	-	−0.14	**−0.27**	−0.04	−0.11	**0.24**	-
**Self-Disclosure**																		
9 L’ Disclosure	-	-	2.70 (0.90)	0.78	-	-	-	-	-	-	-	-	-	**0.49**	0.06	**0.35**	**−0.36**	-
10 E’ Disclosure	-	-	3.48 (1.03)	0.92	-	-	-	-	-	-	-	-	-	-	0.09	**0.29**	**−0.46**	-
**Work Setting**																		
11 WfH Intensity	2.86 (1.21)	-	3.07 (1.53)	-	−0.07	0.06	**0.17**	**0.25**	−0.04	**0.28**	**0.27**	**−0.20**	-	-	-	**−0.19**	−0.08	-
**Communication Aspects**																		
12 Frequency	3.58 (1.14)	-	3.45 (1.05)	-	−0.02	0.09	0.09	**0.18**	0.01	**0.14**	0.12	−0.12	-	-	−0.02	-	**−0.28**	-
13 Barriers	-	-	2.18 (0.92)	0.90	-	-	-	-	-	-	-	-	-	-	-	-	-	-
14 Informal Comm.	3.03 (1.01)	0.92	-	-	0.04	−0.03	**0.14**	**0.23**	−0.02	**0.25**	**0.23**	**−0.20**	-	-	0.10	**0.50**	-	-

Notes. *N* = 227 for study 1 and *N* = 198 for study 2. All correlations are Pearson’s *r*. Significant correlations in boldface (*r* ≥ 0.14 *p* < 0.05; *r* ≥ 0.19 *p* < 0.01; *r* ≥ 0.25 *p* < 0.001). L’ = leader; E’ = employee; WfH = Working from Home; Comm. = communication. ^a^ Gender coded as 0 = male and 1 = female.

**Table 2:** Results of Multiple Linear Regression Analyses predicting employees’ SelfCare behavior in Study 1 (H1, H3a, H5, and H6a), and in Study 2 (H1, H3a, H4a, and H6a), and employees’ disclosure in Study 2 (H2, H3b, H4b, and H6b).

Study 1			Study 2			Study 2		
Predictors	Employees’ SelfCare ^b^		Predictors	Employees’ SelfCare ^b^		Predictors	Employees’ Disclosure	
	β	*SE*		β	*SE*		β	*SE*
Age	0.01	0.02	Age	0.09	0.01	Age	0.12 ^†^	0.01
Gender ^a^	−0.05	0.09	Gender ^a^	0.14 *	0.11	Gender ^a^	0.02	0.13
Leaders’ SelfCare ^b^	0.35 ***	0.07	Leaders’ SelfCare ^a^	0.30 ***	0.07	Leaders’ Disclosure	0.35 ***	0.07
WfH_i	0.19 **	0.04	WfH_i	0.06	0.04	WfH_i	0.03	0.04
Comm. Frequency	0.03	0.04	Comm. Frequency	0.07	0.05	Comm. Frequency	0.06	0.06
Informal Comm.	0.15 *	0.05	Comm. Barriers	−0.12	0.06	Comm. Barriers	−0.41 ***	0.07
L’ SC ^b^ × WfH_i	0.03	0.05	L’ SC ^b^ × WfH_i	−0.04	0.05	L’ D × WfH_i	0.05	0.05
L’ SC ^b^ × CF	−0.04	0.07	L’ SC ^b^ × CF	0.00	0.07	L’ D × CF	−0.08	0.07
L’ SC ^b^ × IC	0.16 *	0.07	L’ SC ^b^ × CB	−0.17 *	0.07	L’ D × CB	−0.27 ***	0.07
***adj. R*** **^2^**	0.27			0.12			0.39	
***N***	227			227			198	

Note. WfH_i = Working from Home intensity; Comm. = Communication; CF = Communication Frequency; IC = Informal Communication; CB = Communication Barriers; L’ SC = Leaders’ SelfCare; L’ D = Leaders’ Disclosure. ^a^ Gender coded as 0 = male and 1 = female. ^b^ subfacet behavior. ^†^ *p* < 0.10, * *p* < 0.05, ** *p* < 0.01, *** *p* < 0.001.

## Data Availability

Data not available for organizational data protection reasons.
